# An unusual case of glomerulonephritis in a patient with non-Hodgkin Mucosal Associated Lymphoid Tissue (MALT) B-cell lymphoma

**DOI:** 10.1186/1471-2369-14-158

**Published:** 2013-07-22

**Authors:** See Cheng Yeo, Khoon Leong Chuah, Hwei Yee Lee, Adrian Liew

**Affiliations:** 1 Department of Renal Medicine, Tan Tock Seng Hospital, Singapore, Singapore; 2 Department of Pathology, Tan Tock Seng Hospital, Singapore, Singapore

**Keywords:** Non-Hodgkin lymphoma, Lupus-like nephritis, Paraneoplastic, Renal failure

## Abstract

**Background:**

Kidney involvement in non-Hodgkin lymphoma is well recognized and glomerulonephritis, when present, has been commonly reported to be associated with a membranoproliferative pattern.

**Case presentation:**

We report a case of a 58-year-old lady with a recurrence of non-Hodgkin MALT B-cell lymphoma, presenting with acute kidney injury, nephrotic range proteinuria and a cellular urinalysis. She underwent a renal biopsy that showed a severe diffuse proliferative and exudative lupus-like glomerulonephritis, which is likely paraneoplastic in nature. We discuss the differential diagnosis and possible pathogenesis of glomerular injury in lymphoma-related proliferative glomerulonephritis.

**Conclusion:**

Differentiating between true lupus nephritis and a paraneoplastic glomerulonephritis is important, as it would have significant implications on treatment and clinical course.

## Background

Kidney involvement in non-Hodgkin lymphoma (NHL) has been well recognized, with reported causes including extranodal lymphoma involvement of the kidneys, acute kidney injury and rarely glomerulonephritis [1-4]. In one of the biggest review of 700 patients with NHL and chronic lymphocytic leukemia with renal manifestations, only 5 and 37 patients with NHL had evidence of renal lymphomatous infiltration and glomerular lesions respectively [1]. Glomerulonephritis, when present, is characterized by the development of proteinuria, [5] with the most common histopathology being membranoproliferative glomerulonephritis (MPGN).

We describe a previously unreported unique case of severe diffuse proliferative and exudative lupus-like glomerulonephritis in a patient with recurrence of non-Hodgkin mucosal associated lymphoid tissue (MALT) B-cell lymphoma.

## Case presentation

### Clinical history and initial laboratory data

A 58-year-old, Chinese, lady with recurrence of non-Hodgkin MALT B-cell lymphoma, was evaluated for the problem of acute kidney injury. She first presented 15 months prior to the current admission with melena and hematemesis. Endoscopic evaluation showed a gastric ulcer on the lesser curvature, overlying a mass lesion, and biopsy confirmed the diagnosis of MALT lymphoma of B-cell origin. Staging of the lymphoma performed classified the disease to be Stage 1, with the tumor limited to the lesser curve of the stomach and sub-centimeter regional lymph nodes. She underwent and completed definitive radiotherapy treatment 3 months after her initial presentation and had been well since. Her last serum creatinine prior to her current presentation was 0.38 mg/dL (34 umol/L). The patient then presented currently with lip swelling and multiple enlarged cervical lymph nodes. Computed tomography revealed multiple enlarged cervical, axillary, mediastinal and external iliac lymph nodes, and excision biopsy of a cervical lymph node confirmed the recurrence of MALT B-cell lymphoma. Initial laboratory investigations demonstrated an elevated serum creatinine of 1.67 mg/dL (148 umol/L), with proteinuria of 4.84 g/day. Urinary microscopic examination was significant for pyuria (181 cells/uL) and microscopic hematuria (57 cells/uL) with coarse granular and hyaline casts. Ultrasound examination of the kidneys revealed normal sized kidneys (left 13.1 cm; right 11.1 cm) with increased echogenicity. Blood leucocyte count was 2,900/uL, hemoglobin was 10.4 g/dL and platelet count was 162,000/uL. All serologic test results for hepatitis B and C, antineutrophil cytoplasmic antibodies and human immunodeficiency virus (HIV) were negative. The antinuclear antibody (ANA) was positive (1:80 titer, homogenous) [Normal < 1/:80 titer] and the anti-double stranded DNA (anti-dsDNA) antibody was mildly elevated (132 IU/mL) [Normal < 25 IU/mL]. Complement levels were normal and serum cryoglobulins, utilizing the qualitative precipitation technique at 4 degrees Celsius, were absent on repeated testing. Serum protein electrophoresis and immunofixation revealed a band in the gamma region, consisting of monoclonal IgG kappa. Serum kappa free light chains was elevated at 49.9 mg/L [Normal: 3.3 - 19.4 mg/L] with a kappa/lambda ratio of 2.35 [Normal: 0.26 - 1.65]. Her renal function continued to deteriorate over a period of one week, with the serum creatinine peaking at 4.07 mg/dL (360 umol/L). A kidney biopsy was performed.

### Kidney biopsy

**Figure 1:**
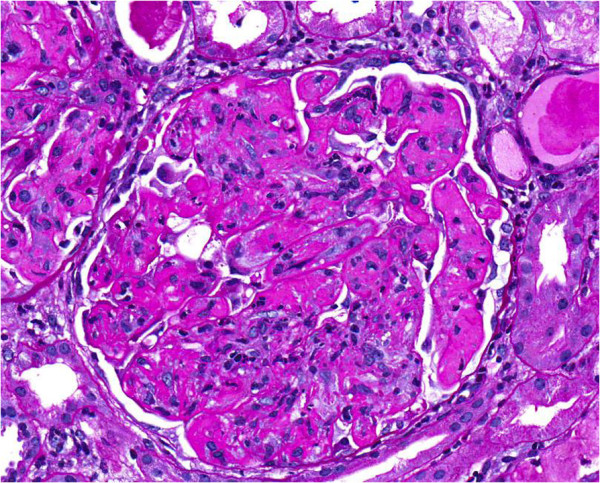
A glomerulus showing hyperlobulation and global narrowing/occlusion of the glomerular capillary lumina with deposits and infiltrating mononuclear leukocytes and occasional neutrophils (periodic acid-Schiff stain; original magnification, ×400).

**Figure 2:**
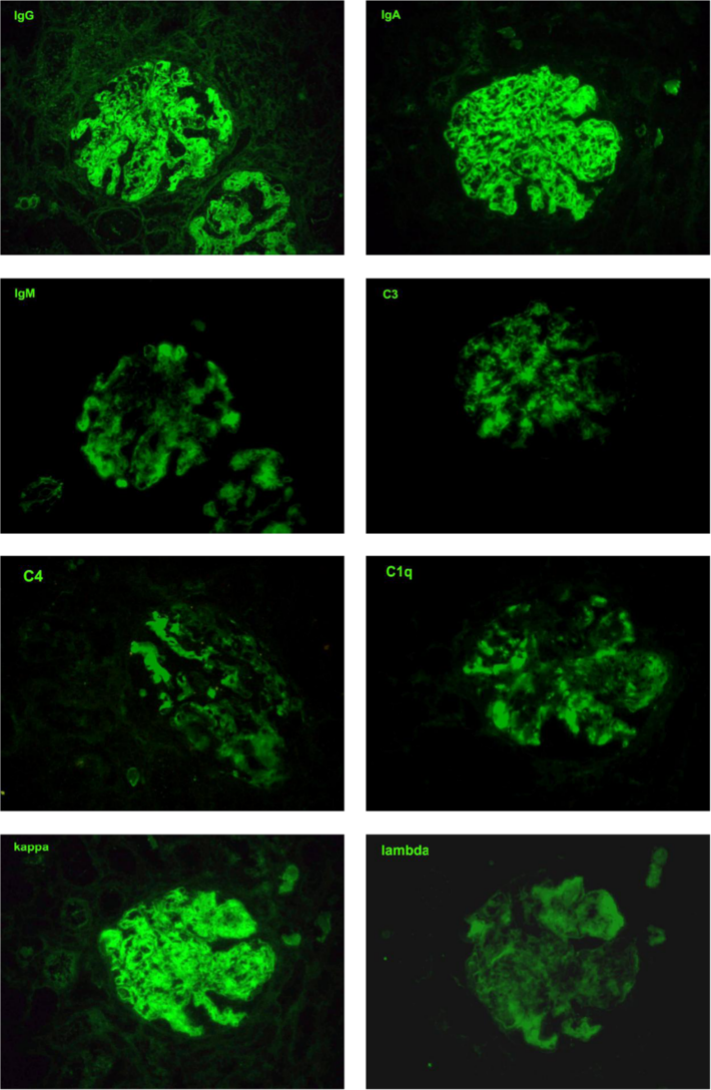
Immunofluorescence photographs showing global positivity in the distribution of the glomerular intraluminal and endothelial deposits for IgG (2+), IgA (2+), IgM (1+), C3 (2+), C4 (1+), C1q (2+), kappa (2+) and lambda (1+) (original magnification, ×400).

**Figure 3:**
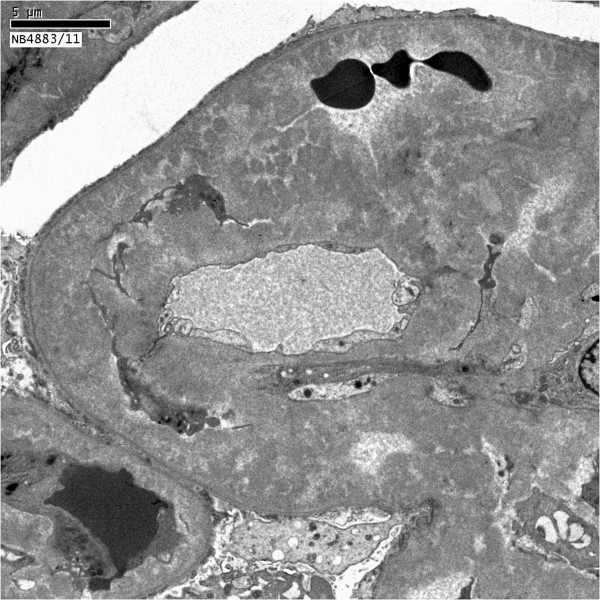
**Electron micrograph showing abundant subendothelial electron dense deposits with intraluminal narrowing.** This appearance corresponds to the global narrowing/occlusion of glomerular capillary lumina by massive eosinophilic deposits together with infiltrating mononuclear leukocytes and neutrophils seen on light microscopy (original magnification × 5000).

**Figure 4:**
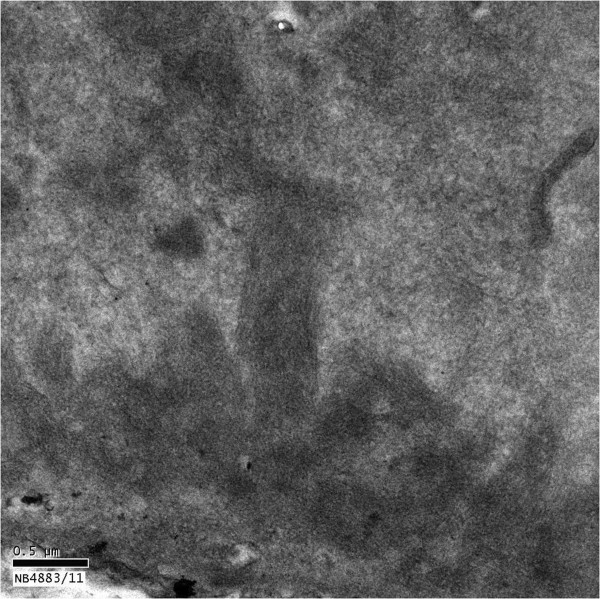
An electron micrograph shows the electron dense deposits with randomly oriented thin fibrils (original magnification, ×40,000).

Of the 42 glomeruli sampled by light microscopy, none were globally sclerotic. The glomeruli appeared markedly enlarged and hyperlobulated, with diffuse and global narrowing or occlusion of the glomerular capillary lumina by eosinophilic deposits associated with infiltrating mononuclear leukocytes and few neutrophils (Figure 1). This was associated with proliferation of mesangial cells and endothelial cell damage and activation. The deposits were massive, forming large intraluminal and subendothelial aggregates that appeared to displace the endothelium. With the silver stain, occasional double contours of glomerular basement membrane were noted. A few of the glomeruli also had nodular mesangial expansion. This material stained eosinophilic, weakly PAS-positive, trichrome-orange and nonargyrophilic. Congo red stain for amyloid was negative. The tubules displayed widespread acute injury with epithelial simplification and interstitial expansion by edema and mild inflammatory infiltrates of mononuclear leukocytes. There were scattered proteinaceous casts. No atypical crystalline casts of the myeloma type or giant cell reaction to cast material were identified. There was mild intimal sclerosis of small and medium-sized arteries and no arteritis was seen. No renal infiltration by lymphoproliferative disorder was identified. Immunofluorescence (Figure 2) showed global positivity in the distribution of the glomerular intraluminal and endothelial deposits for IgG (2+), IgA (2+), IgM (1+), C3 (2+), C4 (1+), C1q (2+), kappa (2+) and lambda (1+); and there was negative staining for albumin and fibrinogen. Electron microscopy showed glomerular capillary lumina that were severely narrowed or occluded by marked infiltration by macrophages and neutrophils associated with subendothelial and intraluminal deposits that appeared moderately electron dense (Figure 3). Most of the deposits had a granular, amorphous texture with the exception of several that contained delicate randomly oriented thin fibrils (Figure 4). Several capillaries also displayed partial mesangial interposition and duplication of glomerular basement membrane enclosing the subendothelial and mesangial electron dense deposits. In some areas, the massive deposits also infiltrate the mesangium associated with mesangiolysis and detachment of mesangial cell processes from the glomerular basement membrane reflection over the mesangium. Foot process effacement was severe, involving over 90% of the total glomerular capillary surface area.

The immunofluorescence findings of intense immune staining support an immune mediated glomerulonephritis. In the setting of widespread B-cell lymphoma with a monoclonal IgG-kappa band, coupled with the more dominant staining of IgG and kappa, the possibility of cryoglobulinemic glomerulonephritis (possibly type 2) containing a monoclonal IgG-kappa component must be considered. Differential diagnosis includes lupus nephritis, which is suggested by the full house staining on the immunofluorescence, which would require clinical confirmation for more definitive diagnosis. The quality of the immune deposits by immunofluorescence and electron microscopy including the presence of some fine fibrillary substructure is more typical of cryoglobulinemic glomerulonephritis, possibly containing a monoclonal IgG-kappa component complexed to IgA. The presence of few thin fibrils within some deposits is not characteristic of fibrillary glomerulonephritis, which typically has thicker fibrils that infiltrate the glomerular extracellular matrix and exhibits dominant immunofluorescence staining for IgG.

### Diagnosis

1. Diffuse endocapillary proliferative and exudative glomerulonephritis with abundant intracapillary and subendothelial immune deposits.

2. Diffuse acute tubular injury.

### Clinical follow-up

Chemotherapy treatment with rituximab, cyclophosphamide, doxorubicin, vincristine and prednisolone (R-CHOP) was initiated. The renal function improved rapidly with just the first cycle of treatment and the serum creatinine improved from a peak of 4.07 mg/dL (360 umol/L) to 0.29 mg/dL (26 umol/L) over a 4-week period. ANA and Anti-dsDNA levels became undetectable immediately after just one round of R-CHOP therapy (Rituximab 513 mg, Cyclophosphamide 822 mg, Doxorubicin 54 mg and Vincristine 1.6 mg; with Prednisolone 100 mg daily for 5 days). Serum cryoglobulins remained consistently absent. However, the patient developed multiple complications from the disease and chemotherapy, including pancytopenia, hospital acquired pneumonia with respiratory failure requiring mechanical ventilation, disseminated herpes infection and severe drug induced cardiomyopathy. The patient subsequently decided for palliative care and declined further chemotherapy after the first cycle of R-CHOP. She was treated symptomatically and discharged home 3 months after her first initial presentation. At the last follow-up in the outpatient clinic, 12 months from her admission, she had a bland urinalysis, no proteinuria and a normal renal function without any treatment.

## Discussion

Glomerulonephritis in the setting of lymphoma is rare and the pattern of glomerular injury varies widely [2]. MPGN has been the most commonly reported histological type of glomerulonephritis, with the prevalence ranging between 14-25% [1,6]. Consequently, MPGN may present in association with cryoglobulinemia or monoclonal gammopathy [5,7]. Other histopathology previously reported includes minimal change disease [4,8], mesangial proliferative IgA nephropathy [9,10] and membranous nephropathy [3,11]. Concurrent glomerulonephritis and direct infiltration of the kidneys by lymphocytic neoplasms has been reported as well, and the pattern of glomerular injury is similar, showing features of MPGN, membranous nephropathy or paraprotein deposition disease [3,4,12]. In one series, Kowalewska et al. showed that 10 (55%) out of 18 patients with renal biopsies demonstrating infiltration of parenchyma by lymphoplasmocytic neoplasms revealed co-existent glomerular injury and suggested that the prevalence of significant glomerulonephritis in this setting may be greater than previously appreciated [12]. It brings to question whether the described associated glomerular disease is paraneoplastic in origin or that of a concurrent primary glomerulonephritis, considering that the occurrence of glomerulonephritis is rare and the morphology of glomerular injury in patients with lymphoma are often heterogeneous [13]. The paraneoplastic nature of these renal lesions may be suspected if spontaneous remission of the glomerular disease follows the successful treatment of the lymphoma. Consequently, the similar heightened immunological activity that exists with lymphoma and glomerulonephritides, resulting in the development of pathological immunoglobulin and immune complexes, could provide a clearer pathophysiologic explanation of the renal lesions observed [6].

In our report, we described a unique case of severe diffuse proliferative and exudative lupus-like glomerulonephritis in the setting of a recurrence of non-Hodgkin MALT lymphoma. Whilst cryoglobulinemic glomerulonephritis was considered as a possibility, the absence of clinical and laboratory evidence of cryoglobulinemia makes it unlikely to be the case. While considering the differential diagnosis of a lupus nephritis, it is noted that MALT lymphoma frequently arises in association with chronic inflammation due to chronic infection or autoimmune disorders [14,15]. Patients with autoimmune disorders, such as systemic lupus erythematosis (SLE) are at increased risk of developing B-cell NHL, including the MALT lymphoma subtype [16-18], with the risk increasing with SLE disease activity [19] and duration of the autoimmune condition [20]. While it is convenient to regard this as due to an unassociated and concomitant proliferative lupus nephritis, it is unlikely after taking into account the clinical course of this patient and the biochemical progression.

The differentiation between a paraneoplastic glomerular disease and a co-existing lupus nephritis is important, as the presence of a severe proliferative lupus nephritis warrants additional induction and maintenance immunosuppression therapy, given on top of the chemotherapy for the lymphoma. On the one hand, our patient had positive autoimmune serologies of ANA and anti-dsDNA, and the immunofluorescence performed on the renal biopsy demonstrated a “full-house” pattern of immunoglobulin and complement deposition within the glomeruli, similar to that seen in proliferative lupus nephritis. However, it is unlikely that the histology is due to an underlying proliferative lupus nephritis for the following reasons:

a. The normal complement levels and low titers of antibodies against anti-dsDNA.

b. The rapid renal response to one cycle of chemotherapy.

Houssiau et al. demonstrated that patients with lupus nephritis have low serum complement C3 and C4 levels and high anti-dsDNA antibodies titers. Indeed, their data suggested that C3 levels and anti-dsDNA titers correlated with the severity of the lupus nephritis at presentation, while anti-dsDNA titers was associated with the severity of the histopathological changes [21]. Hence, if a diffuse proliferative lupus nephritis was responsible for the massive immune deposits and active proliferative glomerular lesions seen on histology in our patient, it would be unusual for the serum complement levels to be normal and the anti-dsDNA titers to be low. Similarly, our patient also lacks signs and symptoms of systemic lupus erythematosus, which further makes the diagnosis of lupus nephritis unlikely.

Ginzler et al [22] looked at the response of serum complement levels and anti-dsDNA titers in patients with lupus nephritis to treatment enrolled in the ALMS study [23]. After 24 weeks of treatment, only 39% and 29% of patients treated with mycophenolate mofetil and cyclophosphamide respectively, had positive anti-dsDNA antibody titers at baseline that subsequently fell to a low or negative titer at endpoint. In our patient, the rapid improvement in renal function and the disappearance of anti-dsDNA serology immediately after only one course of chemotherapy is inconsistent with the clinical tempo of a proliferative lupus nephritis. In contrast, the reduction in tumor load of NHL to only one cycle of R-CHOP therapy has been recognized [24]. This would parallel the resolution of the immunological process leading to the paraneoplastic nature of the proliferative glomerular lesions noted. Likewise, the anti-dsDNA titers would represent a false positive serology arising from polyclonal B-cell activation in the setting of a B-cell lymphoma [25,26], which would become undetectable immediately with treatment of the malignancy.

The pathogenesis of a lupus-like glomerulonephritis in association with lymphoproliferative malignancies remains poorly understood. In the setting of B-cell lymphoma, our patient showed evidence of increased immunoglobulin production that was identified on the serum electrophoresis as monoclonal IgG kappa. This is likely secondary to clonal B-cell expansion due to underlying non-Hodgkin B-cell lymphoma. However, the histology of the renal biopsy revealed abundant immune complex deposition in glomeruli with immunofluorescence showing a “full-house” pattern of immunoglobulin and complement deposits, suggesting the production and deposition of immune complexes arising from polyclonal immunoglobulins. Differential diagnoses of cryoglobulinemia and lupus nephritis were considered but ruled out. The production of polyclonal immunoglobulins and subsequent immune complex formation and deposition within the glomeruli seem to play a central role in the pathogenesis. However, the source of production of polyclonal immunoglobulins is not clear in this case. Furthermore, the unique morphological pattern of “full-house” glomerular immune complex deposition in our patient seems to suggest that the polyclonal immunoglobulin was produced in great abundance and subsequent rapid response to chemotherapy with improvement in renal function suggests that the source of the immunoglobulin production is closely related to the NHL.

## Conclusion

Differentiating between a lupus nephritis and lupus-like diffuse endocapillary proliferative and exudative glomerulonephritis, which is paraneoplastic to a non-Hodgkin MALT B-cell lymphoma is crucial, as treatment is directed to the remission of the lymphoma in the latter. Whilst the renal histopathology suggests a lupus-like proliferative nephritis, the lack of clinical features of systemic lupus erythematosus, the low titers of anti-dsDNA antibodies that are incongruent to the renal disease activity, and the uncharacteristic augmented renal response to only one cycle of chemotherapy are more consistent with a paraneoplastic nature of the glomerular manifestations. Making this distinction will minimize the overuse of immunosuppression in this patient.

## Consent

Written informed consent was obtained from the patient for publication of this case report and any accompanying images. A copy of the written consent is available for review by the Editor of this journal.

## Competing interest

The authors declare that they have no competing interests.

## Authors’ contributions

SCY is involved in the clinical care of the patient and the drafting of the manuscript. CKL and HYL are involved in the processing and interpretation of the renal biopsy, and providing the figures for the manuscript. AL is responsible for the clinical care of the patient, conception of the case report, revision and providing the final approval for the manuscript.

## Pre-publication history

The pre-publication history for this paper can be accessed here:

http://www.biomedcentral.com/1471-2369/14/158/prepub
